# HV1 mtDNA Reveals the High Genetic Diversity and the Ancient Origin of Vietnamese Dogs

**DOI:** 10.3390/ani13061036

**Published:** 2023-03-12

**Authors:** Quan Ke Thai, Thanh-Cong Nguyen, Cong-Trieu Le, Anh-Dung Chung, Tran Minh-Ly Nguyen, Quoc-Dung Tran, Peter Savolainen, Quoc-Dang Quan, Dai-Long Tran, Hoang-Dung Tran

**Affiliations:** 1 Faculty of Natural science Education, Saigon University, 273 An Duong Vuong, Ward 3, District 5, Ho Chi Minh City 72710, Vietnam; 2 Faculty of Biotechnology, Nguyen-Tat-Thanh University, 298A-300A Nguyen-Tat-Thanh Street, District 04, Ho Chi Minh City 72820, Vietnam; 3 Soc Trang Vocational College, 176 Nam Ky Khoi Nghia Street, Ward 7, Soc Trang City 96114, Vietnam; 4 Biotechnology Division, Institute of Agricultural Sciences for Southern Viet Nam, 121 Nguyen Binh Khiem Street, Da Kao Ward, 1st District, Ho Chi Minh City 710302, Vietnam; 5 Faculty of Business Administration, TU Bergakademie Freiberg, Akademiestraße 6, 09599 Freiberg, Germany; 6 Faculty of Biology, University of Education, Hue University, 34 Le 5Loi Street, Hue City 49118, Vietnam; 7 Science for Life Laboratory, School of Biotechnology, Royal Institute of Technology (KTH), 171 65 Solna, Sweden; 8 Agency for Southern Affairs of Ministry of Science and Technology, 31 Han Thuyen Street, Ben Nghe Ward, District 1, Ho Chi Minh City 70055, Vietnam; 9 Department of Supervisor Inspector, Van Lang University, Nguyen Khac Nhu Street, Co Giang Ward, Distric 1, Ho Chi Minh City 700000, Vietnam; 10 Faculty of Biology and Environment, Ho Chi Minh City University of Food Industry, 140 Le Trong Tan Street, Tay Thanh Ward, Tan Phu District, Ho Chi Minh City 72009, Vietnam; 11 Institute of Applied Research and Technology Transfer HUFI, Ho Chi Minh City University of Food Industry, 140 Le Trong Tan Street, Tay Thanh Ward, Tan Phu District, Ho Chi Minh City 72009, Vietnam

**Keywords:** genetic diversity, HV1, mtDNA, haplotype A200, Vietnamese village dog

## Abstract

**Simple Summary:**

This study evaluated the genetic diversity and investigated the origin of Vietnamese domestic dogs on the basis of genetic data. A total of 56 haplotypes (group of genes inherited together from a single parent), including 14 indigenous ones (two of them have not been previously reported) were observed, with some of them implying that Australian dingo and Polynesian dogs originated from Southeast Asian ones, reinforcing the theory of domestication of dogs to the south of the Yangtze River.

**Abstract:**

In this study, samples from 429 dog individuals across three main regions of Vietnam (Southern Vietnam (SVN), Central Vietnam (CVN), and Northern Vietnam (NVN)) were collected to analyze the 582 bp region mtDNA HVI, so as to study the genetic diversity and to screen the rare haplotype E in the Vietnamese village dog population. Nine new haplotypes A, two new haplotypes B, and three haplotypes C were unique to Vietnam dogs, in which the new haplotypes An3, An7, Cn1, and Cn3 concerned mutations at new polymorphism sites (15,517, 15,505, 15,479, and 15,933, respectively) which have not been previously reported. The detection of haplotypes A9 and A29, and the appearance of haplotype A200 in the two individual dogs sampled support that the Southeast Asian dog is the ancestor of today’s Australian dingo and Polynesian dog. The two rare haplotypes E (E1 and E4) were reconfirmed in Vietnamese dogs and discussed. This study also contributes to strengthening the theory of domestication of dogs to the south of the Yangtze River and the Southeast Asian origin of the dingo.

## 1. Introduction

The dog (*Canis familiaris*) is the most popular domesticated animal, with a high morphological and behavioral diversity level. The origin of domestic dogs remains controversial. Leonard et al. (2002) [1] and Savolainen et al. (2002) [2] claimed that dogs originated from Asian Gray Wolves in Southeast Asia and then, according to Wang et al., 2016, migrated from East Asia to the Middle East, Africa, and Europe. Thalmann et al. (2013) [3] pointed to Europe as the primary origin of dogs, while Frantz et al. (2016) reported that dogs might have been domesticated in Eastern and Western Eurasia from distinct wolf populations [4]. 

Studies of the evolutionary origin and relationship among dog breeds in the world using DNA fragments have proven more effective than morphological or archeological tools. The phylogeny and origin of Madagascan dogs [5], native American dog breeds [6], and Tibetan Mastiffs [7] have been determined by analyzing a part or the whole mitochondrial DNA (mtDNA) sequences. The first fully translated Canid (*Canis familiaris*) mitochondrial genome has a 16.7 kb length, similar to vertebrates. The D-loop or control region (CR) (position 15458–16727) is 1270 bp long and plays an essential role in controlling the transcription process. The CR consists of three separate regions, including hypervariable region 1 (HV1), hypervariable region 2 (HV2), and variable tandem repeats [8,9]. HV1 is located at the 5’ end of the CR at position 15,458–16,130 with a length of 673 bp and has a high polymorphism rate, as in human beings, which is usually used in forensics in the case of lacking or seriously defective DNA samples. HV1 is also used to identify SNPs (single-nucleotide polymorphisms) and determine village dog haplotypes [2,10]. HVI can be grouped into six haplogroups: A, B, C, D, E, and F [10]. The three common haplogroups, A, B, and C, have a widespread worldwide distribution of 71.3%, 17.26%, and 7.80%, respectively. In contrast, the distribution of haplogroups D, E, and F is more geographically specific; for example, haplogroup D can only be found in particular regions such as Turkey, Spain, and Scandinavia; haplogroup E can only be found in mountainous Japan, China, and South Korea; haplogroup F can only be found in snowy areas of Japan and Siberia [6,10].

In Vietnam, especially in rural regions, village dogs are usually free-ranging and free-breeding. In addition to village dogs with standard slim body shapes, there are some groups of dogs with a unique appearance, such as the Phu Quoc ridgeback dog (from Phu Quoc Island, South of Vietnam), Hmong bobtail dog (from Lao Cai, Son La province, North of Vietnam), and Bac Ha dog (from Lao Cai province, North of Vietnam). The indigenous dogs in Vietnam are highly adapted to the local environmental conditions and possess diversified morphological and behavioral characteristics. The origin and diversity of Vietnamese dog breeds remain uncharacterized. In initial research evaluating the genetic diversity of Phu Quoc dogs [11], we found the rare haplogroup E (over 10%) in addition to the ubiquitous haplogroups A, B, and C in the population [12]. The occurrence of haplogroup E in Vietnamese dogs, previously found only in Japanese and Korean dogs, has raised a question about the distribution of this haplogroup in Vietnam and whether other breeds in Vietnam have haplogroup E or not. 

This study analyzed the mtDNA HV1 region of 429 individuals (397 newly collected individuals and 32 individuals from previously published papers) to evaluate the genetic diversity of the Vietnamese dog population. We also screened haplotype E in the Vietnamese dog population. The relationship among Vietnamese diverged indigenous dog breeds and with other dog populations such as those in Thailand, Iran, Turkey, central China, and southern of China [6,10,11] is also investigated in this study. 

## 2. Materials and Methods

### 2.1. Sample Collection

**Figure 1:**
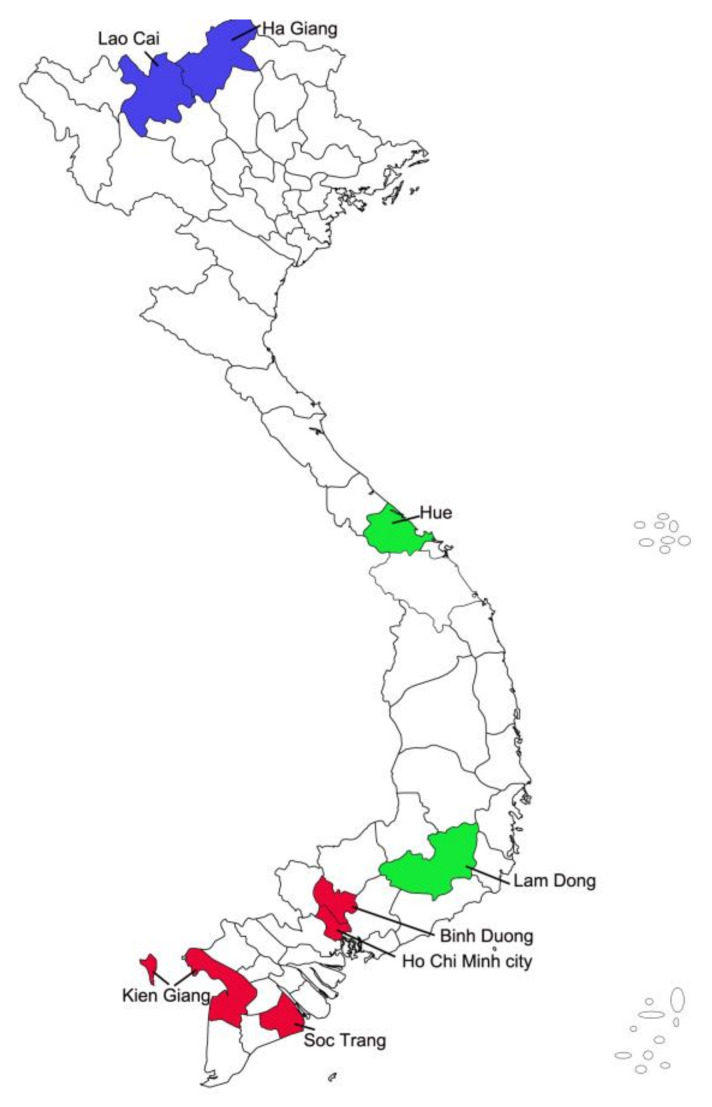
Sampling sites. SVN (red): 310 samples (Kien Giang: 223, Soc Trang: 21, Binh Duong: 7, Ho Chi Minh city: 59); CVN (green) 41 samples (Lam Dong: 26, Hue: 15); NVN (blue): 46 samples (Lao Cai: 15, Ha Giang: 31).

Dog hairs were sampled across the primary regions of Vietnam: Southern Vietnam (SVN), Central Vietnam (CVN), and Northern Vietnam (NVN). To avoid testing related dogs, samples from sibling dogs or dogs with a maternal bond were excluded. Hair samples were stored separately in a labeled plastic zip-lock bag at −30 °C, while blood samples were stored in EDTA-containing falcon tubes at 4 °C. Thirty-two samples from the previously published literature (totaling 429 samples) were analyzed in this study. Of these, 417 samples with geographical information were classified into SVN, CVN, and NVN groups; 12 samples (from literature) lacking geographical information were grouped into “other”. In these samples, 100 Phu Quoc ridgeback dogs were sampled in Ho Chi Minh City and Phu Quoc island, which are in Southern Vietnam (Figure 1).

### 2.2. DNA Extraction

DNA from hair roots was extracted using a previously reported protocol [13]. Forty hair roots of each dog were soaked and vortexed with lysis buffer (Tris-HCl 10 mM, pH 8; EDTA 10 mM, Triton X-100 1%, SDS 1%) for 30 s and incubated at 50 °C for 20 min. The solution was then incubated with 5 μL of proteinase K (20 mg/mL) at 50 °C for 1 h for protein digestion. Lysed DNA was isolated using a phenol:chloroform:isoamyl alcohol mixture and precipitated in absolute ethanol with NaCl 0.2 M. DNA was then dissolved in water and stored at −30 °C for further analysis. DNA from blood samples was extracted using the ISOLATE II Genomic DNA Kit (Meridian Bioscience, OH, US, Cat.no. BIO-52066). The quantitation and purity of DNA samples were estimated using absorbance measurements and agarose gel electrophoresis methods. 

### 2.3. DNA Amplification

The 1267 bp CR fragment was amplified using two primers: 15412F, 5′–CCACTATCAGCACCCAAAG–3′ and 16625R, 5′–AGACTACGAGACCAAATGCG–3′ [14]. The PCR reaction mixture consisted of 2 µL of template DNA, 0.05 U/µL of Taq DNA Polymerase, 0.2 mM of each dNTP, 0.2 mM MgSO_4_, 10 mM KCl, 8 mM (NH_4_)_2_SO_4_, 10 mM Tris-HCl (pH 8.8), and 10 µM of forward (15412F) and reverse (16625R) primers in a total volume of 20 µL. After a denaturation step (95 °C, 5 min), the amplification was performed in a Techne TC-3000 (USA) thermocycler for 35 cycles (denaturation at 95 °C for 30 s, primer annealing at 49 °C for 30 s, extension at 72 °C for 1 min), finalized by an extension step at 72 °C for 5 min. Three microliters of PCR products were analyzed on a 1% agarose gel with ethidium bromide staining. 

### 2.4. DNA Sequencing

The sequencing of PCR products was carried out by NICEM, South Korea, using the Sanger method [15] with two primers (15412F, CCACTATCAGCACCCAAAG and 16114R, CCTGAAACCATTGACTGAATAG) [14]. The result of sequencing was evaluated using FinchTV 1.4.0. Manual editing was performed to reconcile inconsistencies between forward and reverse sequences. All sequences were aligned with reference sequence [9] and trimmed using ClustalW [16] and FinchTV1.4.0 to produce 582 bp sequences. 

### 2.5. Haplotyping DNA Sequencing

HV1 haplotypes of 582 bp sequences were identified (Supplementary Materials–Table S1) using the Haplotype Identifier [17]. Three hundred ninety seven DNA sequences of Vietnamese dogs in this study were deposited in GenBank under accession nos. MG799222–MG799321, MG793253–MG793352 and OQ241527–OQ241723 (Supplementary Materials–Table S2).

### 2.6. Data Analysis DNA Sequencing

Data from this study (397 individuals) and previously published data (32 Vietnamese individuals [5,10]) resulted in a sample set of 429 dogs for analysis. Genetic diversity measures (nucleotide diversity, haplotype diversity, and average nucleotide difference), AMOVA, and genetic distances among dog populations were estimated as F_ST_ values using Arlequin 3.5.2.2 [18]. Minimum-spanning networks were drawn manually according to the suggestion of Arlequin 3.5.2.2. To compare the number of haplotypes among populations, resampling with replacement was implemented with the sample size adjusted to 44 (the smallest population, i.e., CVN dogs) and 1000 replications, using in-house developed software.

## 3. Results

A total of 397 DNA samples of Vietnamese dogs were sequenced, giving the base compositions C—27.3%, T—29.99%, A—26.9%, and G—15.81%, similar to previously reported studies on this region [2,5,10,19,20]. The similarity in the base composition and the sequence alignment confirmed the mtDNA origin of sequenced DNAs. 

**Figure 2:**
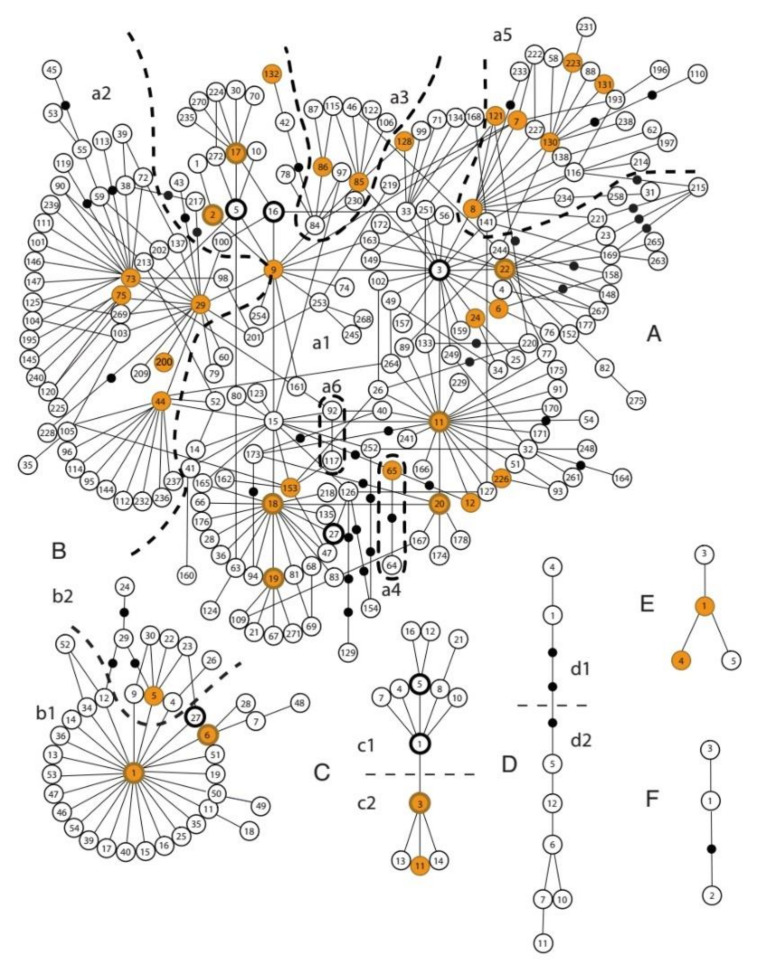
Minimum spanning networks showing Vietnam village dog haplotypes (yellow-filled circles) in the worldwide dog haplotypes (blank circles). The haplogroups A, B, C, D, E, and F are represented by the letters A, B, C, D, E, and F, respectively.

There were 56 haplotypes found in the analyzed dogs. These haplotypes belonged to four different haplogroups: A, B, C, and E. Interestingly, haplotypes E1 and E4, rare in dogs worldwide, were found with high frequency in Vietnamese dogs (8.2%). Except for one dog without geographical information, all 34 remaining dogs harboring haplotype E were in SVN. Of these 56 haplotypes, nine new haplotypes A, two new haplotypes B, and three haplotypes C were unique to Vietnam dogs. In contrast, most new haplotypes were the new combination of known mutations; the new haplotypes An3, An7, Cn1, and Cn3 concerned mutations at new polymorphism sites (15,517, 15,505, 15,479, and 15,933, respectively), which have not been previously reported. Most dogs (298/429) harbored a particular haplotype that is universally found across the world (universal type—UT) (233/429) or that differed by one mutation from the UT (UT-derived—UTd) (65/429) (Table 1). Only two of the 10 universal types of haplotypes (B1 and C2) were shared in these three groups. Haplotypes in Vietnamese village dogs were distributed in nearly all haplogroups A, B, C, and E, i.e., a1, a2, a3, a4, a5, b1, b2, c2, and E (Figure 2).

With its high genetic diversity, the Vietnamese village dog population fits well with the global picture of canine mtDNA haplotype diversity. In line with the origin of dog domestication, dogs in the South of the Yangtze River [10], Thailand [21], and Vietnam have high haplotype diversity with a high number of haplotypes distributed in most sub-haplogroups. The number of sub-haplogroups decreases in the dog populations from the origin to other regions, with only five in southwest Asia and four in Europe [21]. The haplotype composition of other dog populations reported was collected to calculate haplotype and nucleotide diversity (Table 2). This analysis also showed the high genetic diversity of Thai and Vietnamese populations compared with other dog populations in the western region of Asia and Europe. 

The haplotype diversity in NVN was high due to its location near the Yangtze River, and these values decreased gradually in south regions (0.9219 ± 0.0242, 0.9154 ± 0.0327, and 0.8994 ± 0.0079 for NVN, CVN and SVN, respectively). Although the number of haplotypes found in SVN was relatively higher than those in CVN and NVN, the re-sampling with the size 44 showed that the number of haplotypes was low (15.16 ± 1.28) while those in CVN and NVN were around 23. Interestingly, while the haplotype diversity decreased from north to south, the nucleotide diversity in NVN (0.009830 ± 0.005304) was explicitly lower than that in CVN and SVN (0.015095 ± 0.007880 and 0.014596 ± 0.007486, respectively). Thus, it can be said that dogs in the NVN are genetically close to each other, and the gene pool is in relative equilibrium.

## 4. Discussion

The NVN founder population had a high level of genetic diversity, and the descendant haplotypes arose mainly via in-breeding over time, as evidenced by their relationship. Alternatively, the genetic structure of the CVN and SVN dog populations was more complex. From the origin of domestication, the migration of dogs from north to south gave lower haplotype diversity in the dog populations of these regions. However, there were seemly some events in these regions resulting in the incorporation of new haplotypes (in other haplogroups) into the populations. Although newly haplotypes were seemingly not able to not compensate for the loss of haplotypes during the migration, the haplotypes from other haplogroups (i.e., haplogroup E, apart from haplogroup A and C) and the fixation of new haplotypes in the population increased the nucleotide diversity. It can be hypothesized that (1) new E haplotypes were subjected to the SVN populations by inter-breeding haplotype E–harboring female wolves and male dogs during the migration, or (2) haplotype E-harboring dogs were imported into the SVN dog populations. There is strong and weak support for each hypothesis. The former could explain the high rate of haplotype E in the SVN population, but the existence of wolves in Vietnam has not been confirmed so far. The latter can be readily accepted since the haplotype E-harboring dogs have also been found somewhere in the world [25], but the high rate of haplotype E in the populations needs a reasonable explanation.

All over the world, three haplogroups (A, B, and C) are widely distributed within dog populations. In contrast, haplogroups D, E, and F are rare groups with less than 3%. Like haplogroups D and F, haplogroup E is distributed in relatively narrow areas such as Siberia, Japan, Korea, Indonesia, Thailand, and Vietnam [10,12,25,26]. More specifically, most haplotype E–harboring dogs are in Kien Giang province (Rach Gia city, Phu Quoc Island) and Ho Chi Minh city. While the proportion of haplogroup E ranges from 3.33% to 7.5% in Shiba, Jindo, Pungsan, and Thai VD, it is incredibly high in Vietnamese VD with 8.2% (Table 3). 

Li and Zang studied the origin and evolution of 50 Tibetan Mastiffs and their relationship with other dog breeds worldwide by analyzing 582 bp sequences in the HV1. In their analyses, only 1.78% of HV1 sequences were found harboring haplogroup D, along with 0.63% harboring haplogroup E and 0.19% harboring haplogroup F. These haplogroups are rare and believed to be the result of post-domestication wolf–dog hybridization [27]. Imes et al. (2012) sequenced ~16.7 kb long of the whole canine mitochondrial genome of 100 unrelated domestic dogs. Results showed 35 haplotypes clustering within one of the four haplogroups (A, B, C, and D) described above. Of these, there were 23 haplotypes previously observed, along 11 new haplotypes and an ambiguous one. The 10 most frequently observed haplotypes (A2, A11, A16, A17, A18, A19, A22, A26, B1, and C3) contributed approximately 53% of the sequences [28]. A study on Malagasy dogs found that 100% of examined samples harbored haplogroups A, B, and C. In some previous studies, Thai Ridgeback dogs were also included [10,26], which showed that all their HV1 sequences (seven samples) belonged to the haplogroups A and B.

**Figure 3:**
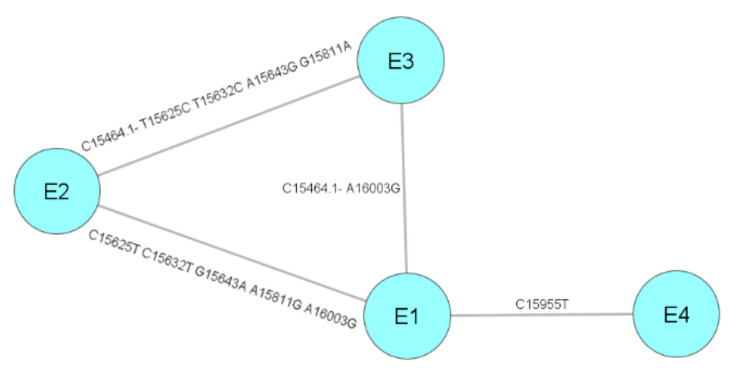
Minimum spanning network of haplotypes in haplogroup E.

As in the case of haplogroups D and F, the number of haplotypes in haplogroup E was minimal with four different haplotypes; thus, the introduction of haplotype E into the domestic dog population seems to have occurred long after haplogroups A, B, and C. Despite belonging to haplogroup E, haplotype E2 was found only in one individual (Siberian Laika dog) in Siberia, with five nucleotides different from the two closely related haplotypes E1 and E3 (Figure 3). This haplotype E2 probably resulted from an independent dog–wolf crossbreeding event when the domestic dog migrated to Siberia. The detection of domestic dogs harboring haplotype E1 and E4 in Thailand and Vietnam, with dogs harboring haplotype E1 only being found in East Asia suggested that the origin of E1/E4 haplotype dogs is in Southeast Asia. This dog–wolf crossbreeding could have occurred at the origin of domestication or during the migration of domestic dogs from the original domestication place to the south. Crossbreeding between wolves and dogs has happened many times, giving an admixture with wolves of about 10% [29]. It can be considered that later crossbreeding should result in dogs being genetically closer to wolves. Hence, these haplotype E-harboring dogs are genetically closer to wolves than other dogs. However, further studies on different aspects (such as genetics and behavioral characteristics) of this hypothesis should be performed.

In Vietnamese village dogs, haplotypes belonging to haplogroups B, C, and E did not have high diversity (two or three haplotypes). In contrast, most of the recorded haplotypes belonged to haplogroup A. The network of haplotypes belonging to haplogroup A shows that the haplotype diversity of the Vietnamese village dog was high. However, most haplotypes were close to each other, differing only by one or more nucleotides from the nearest haplotype, except An1, which differed by three nucleotides from the nearest haplotype. This indicated that the diversity of the current Vietnamese haplogroup A-harboring dog population was formed by mutations occurring within the population during evolution, rarely introduced from other populations. Notably, haplotypes A9 and A29 were detected in Vietnamese domestic dogs. These are two haplotypes found in dingoes, in which dogs carrying haplotype A29 are considered the ancestral haplotype of today’s dingoes.

Another critical point is that two ancient haplotypes, Arc1 and Arc2, were also recorded among Vietnamese domestic dogs through haplotypes A9, A7, A8, and A75. Due to DNA degradation over time in the archaeal samples, the Arc1 and Arc2 haplotypes were only identified on the basis of nucleotide sequences from positions 15,458 to 15,720 of the HV1 region. Compared with the haplotypes in the current dog population, haplotype Arc1 may be equivalent to haplotypes A3, A5, A6, A7, A8, and A9 because these haplotypes have the same sequence region from positions 15,458 to 15,720 and are similar to Arc1. Similarly, Arc2 is equivalent to haplotypes A75, A120, A192, and A194. The differences between these haplotypes are outside the nucleotide sequence region 15,458–15,720. This analysis result is entirely consistent with the previous study by Oskarsson et al. [26], confirming that the Southeast Asian dog is the ancestor of today’s Australian dingo and Polynesian dog, which is also further evidence for the ancient origin of Vietnamese village dogs. The new finding in this study was the appearance of haplotype A200 in the two individual dogs sampled in two provinces in CVN (Lam Dong and Hue province), which has previously only been detected in the dingo [2]. This similarity could lead to the hypothesis that the Vietnamese domestic dog was the first dog to migrate to Australia, forming the dingo population. However, more complete studies are needed with dog samples collected from other parts of Southeast Asia to confirm this hypothesis.

## 5. Conclusions

Vietnam village dogs have a high level of haplotypes A, B, and C, as well as the rare E haplogroup; the nucleotide diversity compared to other dog breeds in the world, together with the close relationship between haplotypes, indicates high genetic diversity and their ancient origin. The study contributes to strengthening the theory of the domestication of dogs to the south of the Yangtze River and the Southeast Asian origin of the dingo. In addition, the detection of haplotype A200 in the Vietnamese domestic dog population also provides a more detailed hypothesis about the origin of the dingo.

## Figures and Tables

**Table 1:** Diversity statistics for Vietnamese dogs.

Regions	ABC (DEF)	nA (%)	nB (%)	nC (%)	nE (%)	nHT	nHTres44	HTuq	Haplotype Diversity	Nucleotide Diversity	Mean of Pairwise Differences
**Total**	394 (35)	232 (54.1)	97 (22.6)	65 (15.2)	35 (8.2)	56	18.96 (± 2.32)	14	0.9210 ± 0.0065	0.014819 ± 0.007586	8.639566 ± 3.998569
**SVN**	284 (34)	153 (48.1)	75 (23.6)	56 (17.6)	34 (10.7)	31	15.16 (± 1.28)	3	0.8994 ± 0.0079	0.014596 ± 0.007486	8.509454 ± 3.945443
Rach Gia	78 (5)	49 (59)	15 (18.1)	14 (16.9)	5 (6)	18		1 *			
Ha Tien	15 (0)	8 (53.3)	5 (33.3)	2 (13.3)	0 (0)	7		0			
Phu Quoc	118 (15)	56 (42.1)	39 (29.3)	23 (17.3)	15 (11.3)	17		1 *			
Soc Trang	21 (0)	12 (57.1)	4 (19)	5 (23.8)	0 (0)	14		1			
Binh Duong	7 (0)	6 (85.7)	1 (14.3)	0 (0)	0 (0)	4		0			
Ho Chi minh city	45 (14)	22 (37.3)	11 (18.6)	12 (20.3)	14 (23.7)	17		1			
**CVN**	44 (0)	23 (52.3)	15 (34.1)	6 (13.7)	0 (0)	23	23	5	0.9154 ± 0.0327	0.015095 ± 0.007880	8.800211 ± 4.137619
Lam Dong	26 (0)	12 (46.2)	9 (34.6)	5 (19.2)	0 (0)	12		2 *			
Hoi An	3 (0)	2 (66.7)	0 (0)	1 (33.3)	0 (0)	12		0			
Hue	15 (0)	9 (60)	6 (40)	0 (0)	0 (0)	3		3			
**NVN**	55 (0)	50 (90.9)	3 (5.5)	2 (3.6)	0 (0)	26	22.4 (± 1.41)	7	0.9219 ± 0.0242	0.009830 ± 0.005304	5.721212 ± 2.782501
Sapa	9 (0)	9 (100)	0 (0)	0 (0)	0 (0)	7		0			
Bac Ha	15 (0)	14 (93.3)	1 (6.7)	0 (0)	0 (0)	11		2			
Hmong	31 (0)	27 (87.1)	2 (6.5)	2 (6.5)	0 (0)	15		5			
**Other**	11 (1)	6 (50)	4 (33.3)	1 (8.3)	1 (8.3)	8		0			

ABC (DEF)**:** the number of sequences belonging to the haplogroups A, B, and C (E, D, and F); nA (%), nB (%), nC (%), nE (%): the number of sequences (the percentages) belonging to haplogroups A, B, C, and E; nHT: number of haplotypes; nHTres44: number of haplotypes with sample size adjusted to 44; HTuq: * share one unique haplotype Cn2.

**Table 2:** Haplotype diversity and nucleotide diversity of examined dog populations.

Dog Population	Haplotype Diversity	Nucleotide Diversity
Serra da Estrela [19]	0.8520 ± 0.0299	0.016842 ± 0.008789
Portuguese sheepdog [19]	0.4841 ± 0.1094	0.008291 ± 0.004644
German Shepherd [22]	0.6842 ± 0.0917	0.008681 ± 0.004928
Maltese dog [23]	0.8046 ± 0.0697	0.011758 ± 0.006335
Kangal [2,20]	0.8407 ± 0.0274	0.015272 ± 0.007852
Tibetan mastiff [10,22]	0.8063 ± 0.0181	0.006645 ± 0.003724
Shiba [2,24]	0.8161 ± 0.0447	0.012221 ± 0.006563
Jindo [2,10]	0.7308 ± 0.0640	0.006645 ± 0.003762
Pungsan [2,10]	0.9064 ± 0.0244	0.011367 ± 0.006090
Thai dog [2,10]	0.9493 ± 0.0124	0.009599 ± 0.005145
Vietnamese dog	0.9210 ± 0.0065	0.014819 ± 0.007586

**Table 3:** Haplotype and nucleotide diversity of haplotype E in examined dog populations.

Population	n	No. of Haplotype E Individuals	No. of Individuals
Jindo	53	1	3 (6%)
Pungsan	40	1	3 (7.5%)
Shiba	30	1	1 (3.33%)
Thai village dog	105	2	4 (3.8%)
Vietnamese village dog	429	2	35 (8.2%)

## Data Availability

Sequences used in this study are available in the GenBank with accession nos. MG799222—MG799321, MG793253—MG793352 and OQ241527—OQ241723.

## Supplementary Materials

The following supporting information can be downloaded at https://www.mdpi.com/article/10.3390/ani13061036/s1: Table S1. List of Vietnamese dog haplotypes. Table S2. GenBank accession number of Vietnamese dogs’ HV1 sequences.

## References

[1] Leonard J.A., Wayne R.K., Wheeler J., Valadez R., Guillen S., Vila C. (2002). Ancient DNA evidence for Old World origin of New World dogs. Science.

[2] Savolainen P., Zhang Y.P., Luo J., Lundeberg J., Leitner T. (2002). Genetic evidence for an East Asian origin of domestic dogs. Science.

[3] Thalmann O., Shapiro B., Cui P., Schuenemann V.J., Sawyer S.K., Greenfield D.L., Germonpre M.B., Sablin M.V., Lopez-Giraldez F., Domingo-Roura X. (2013). Complete mitochondrial genomes of ancient canids suggest a European origin of domestic dogs. Science.

[4] Frantz L.A., Mullin V.E., Pionnier-Capitan M., Lebrasseur O., Ollivier M., Perri A., Linderholm A., Mattiangeli V., Teasdale M.D., Dimopoulos E.A. (2016). Genomic and archaeological evidence suggest a dual origin of domestic dogs. Science.

[5] Ardalan A., Oskarsson M.C.R., Asch B.V., Rabakonandriania E., Savolainen P. (2015). African origin for Madagascan dogs revealed by mtDNA analysis. R. Soc. Open Sci..

[6] Van Asch B., Zhang A.-B., Oskarsson M.C.R., Klütsch C.F.C., Amorim A., Savolainen P. (2013). Pre-Columbian origins of Native American dog breeds, with only limited replacement by European dogs, confirmed by mtDNA analysis. Proc. R. Soc. Lond. B Biol. Sci..

[7] Li Q., Liu Z., Li Y., Zhao X., Dong L., Pan Z., Sun Y., Li N., Xu Y., Xie Z. (2008). Origin and phylogenetic analysis of Tibetan Mastiff based on the mitochondrial DNA sequence. J. Genet. Genom..

[8] Bekaert B., Larmuseau M.H.D., Vanhove M.P.M., Opdekamp A., Decorte R. (2012). Automated DNA extraction of single dog hairs without roots for mitochondrial DNA analysis. Forensic Sci. Int. Genet..

[9] Kim K.S., Lee S.E., Jeong H.W., Ha J.H. (1998). The complete nucleotide sequence of the domestic dog (*Canis familiaris*) mitochondrial genome. Mol. Phylogenetics Evol..

[10] Pang J.-F., Kluetsch C., Zou X.-J., Zhang A.-B., Luo L.-Y., Angleby H., Ardalan A., Ekstrom C., Skollermo A., Lundeberg J. (2009). mtDNA data indicate a single origin for dogs south of Yangtze River, less than 16,300 years ago, from numerous wolves. Mol. Biol. Evol..

[11] Hillbertz N.H.C.S. (2007). The origin of the ridge and associated anomalies in Rhodesian Ridgebacks.

[12] Quan T.K., Tu N.V., Trinh T.N., Hieu H.V., Dung C.A., Dung T.H. (2016). Evaluation of genetic diversity of Phu Quoc ridgeback dogs based on mitochondrial DNA Hypervariable-1 region. Vietnam J. Biotechnol..

[13] Quan T.K., Tu N.V., Hieu H.V., Cong N.T., Dung T.H. (2016). A simple protocol for DNA extraction from dog hairs. Vietnam J. Biol..

[14] Gundry R.L., Allard M.W., Moretti T.R., Honeycutt R.L., Wilson M.R., Monson K.L., Foran D.R. (2007). Mitochondrial DNA analysis of the domestic dog: Control region variation within and among breeds. J. Forensic Sci..

[15] Sanger F., Nicklen S., Coulson A.R. (1977). DNA sequencing with chain-terminating inhibitors. Proc. Natl. Acad. Sci. USA.

[16] Larkin M.A., Blackshields G., Brown N.P., Chenna R., McGettigan P.A., McWilliam H., Valentin F., Wallace I.M., Wilm A., Lopez R. (2007). Clustal W and Clustal X version 2.0. Bioinformatics.

[17] Thai Q.K., Chung D.A., Tran H.-D. (2017). Canis mtDNA HV1 database: A web-based tool for collecting and surveying Canis mtDNA HV1 haplotype in public database. BMC Genet..

[18] Excoffier L., Lischer H.E.L. (2010). Arlequin suite ver 3.5: A new series of programs to perform population genetics analyses under Linux and Windows. Mol. Ecol. Resour..

[19] Van Asch B., Pereira L., Pereira F., Santa-Rita P., Lima M., Amorim A. (2005). MtDNA diversity among four Portuguese autochthonous dog breeds: A fine-scale characterisation. BMC Genet..

[20] Koban E., Gökçek Saraç Ç., Açan S.C., Savolainen P., Togan İ. (2009). Genetic relationship between Kangal, Akbash and other dog populations. Discret. Appl. Math..

[21] Zhang L., Liu Y., Thai Ke Q., Ardalan A., Boonyaprakob U., Savolainen P. (2020). Complete range of the universal mtDNA gene pool and high genetic diversity in the thai dog population. Genes.

[22] Ren Z., Chen H., Yang X., Zhang C. (2017). Phylogenetic analysis of Tibetan mastiffs based on mitochondrial hypervariable region I. J. Genet..

[23] Takahasi S., Miyahara K., Ishikawa H., Ishiguro N., Suzuki M. (2002). Lineage classification of canine inheritable disorders using mitochondrial DNA haplotypes. J. Vet. Med. Sci..

[24] Okumura N., Ishiguro N., Nakano M., Matsui A., Sahara M. (1996). Intra- and interbreed genetic variations of mitochondrial DNA major non-coding regions in Japanese native dog breeds (*Canis familiaris*). Anim. Genet..

[25] Klutsch C.F., Seppala E.H., Fall T., Uhlen M., Hedhammar A., Lohi H., Savolainen P. (2011). Regional occurrence, high frequency but low diversity of mitochondrial DNA haplogroup d1 suggests a recent dog-wolf hybridization in Scandinavia. Anim. Genet..

[26] Oskarsson M.C., Klutsch C.F., Boonyaprakob U., Wilton A., Tanabe Y., Savolainen P. (2012). Mitochondrial DNA data indicate an introduction through Mainland Southeast Asia for Australian dingoes and Polynesian domestic dogs. Proc. R. Soc. B.

[27] Li Y., Zhang Y. (2012). High genetic diversity of Tibetan Mastiffs revealed by mtDNA sequences. Chin. Sci. Bull..

[28] Imes D.L., Wictum E.J., Allard M.W., Sacks B.N. (2012). Identification of single nucleotide polymorphisms within the mtDNA genome of the domestic dog to discriminate individuals with common HVI haplotypes. Forensic Sci. Int. Genet..

[29] Wang G.-D., Zhai W., Yang H.-C., Wang L., Zhong L., Liu Y.-H., Fan R.-X., Yin T.-T., Zhu C.-L., Poyarkov A.D. (2016). Out of southern East Asia: The natural history of domestic dogs across the world. Cell Res..

