# Supplementary material for: HV1 mtDNA Reveals the High Genetic Diversity and the Ancient Origin of Vietnamese Dogs
Source: Animals (Basel). 2023 Mar 12;13(6):1036. doi: 10.3390/ani13061036 (PMC10044065; doi:10.3390/ani13061036)
Supplement: Supplementary file 1 [file animals-13-01036-s001.zip › animals-2190968-supplementary.pdf]

Table S1: List of Vietnamese dog haplotypes

	Haplotype	SVN	CVN	NVN	Other	Total
1	A2	3	1	0	0	4
2	A6	0	1	0	0	1
3	A7	4	0	0	0	4
4	A8	1	2	1	1	5
5	A9	2	2	0	0	4
6	A11	51	3	0	2	56
7	A12	0	0	1	0	1
8	A13	0	1	0	0	1
9	A17	18	2	0	1	21
10	A18	29	1	0	0	30
11	A19	9	0	0	0	9
12	A20	1	1	0	0	2
13	A22	0	0	1	0	1
14	A24	2	0	0	0	2
15	A29	0	2	4	0	6
16	A44	0	1	4	0	5
17	A65	13	0	1	0	14
18	A73	7	1	13	1	22
19	A75	2	0	0	0	2
20	A85	1	0	2	0	3
21	A86	0	0	1	0	1
22	A121	1	0	7	0	8
23	A128	0	0	2	0	2
24	A130	0	0	1	0	1
25	A131	0	0	2	0	2
26	A132	4	0	0	1	5
27	A140	0	0	1	0	1
28	A143	0	0	1	0	1
29	A153	2	0	0	0	2
30	A200	0	2	0	0	2
31	A223	3	0	0	0	3
32	A226	0	0	1	0	1
33	An1	0	1	0	0	1
34	An2	0	0	1	0	1
35	An3	0	0	1	0	1
36	An4	0	0	1	0	1
37	An5	0	0	1	0	1
38	An6	0	0	2	0	2
39	An7	0	0	1	0	1
40	An8	0	1	0	0	1
41	An9	0	1	0	0	1
42	B1	66	12	1	4	83
43	B3	1	0	0	0	1
44	B5	2	1	1	0	4

	Haplotype	SVN	CVN	NVN	Other	Total
45	B6	4	0	0	0	4
46	B10	2	1	0	0	3
47	Bn1	0	0	1	0	1
48	Bn2	0	1	0	0	1
49	C2	30	4	2	0	36
50	C3	21	1	0	1	23
51	C11	1	0	0	0	1
52	Cn1	1	0	0	0	1
53	Cn2	2	1	0	0	3
54	Cn3	1	0	0	0	1
55	E1	20	0	0	1	21
56	E4	14	0	0	0	14
					**Total:**	**429**

Table S2: GenBank accession number of Vietnamese dogs' HV1 sequences

No.	Sample	GenBank accession number
1	VD1	MG799222
2	VD2	MG799223
3	VD3	MG799224
4	VD4	MG799225
5	VD5	MG799226
6	VD6	MG799227
7	VD7	MG799228
8	VD8	MG799229
9	VD9	MG799230
10	VD10	MG799231
11	VD11	MG799232
12	VD12	MG799233
13	VD13	MG799234
14	VD14	MG799235
15	VD15	MG799236
16	VD16	MG799237
17	VD17	MG799238
18	VD18	MG799239
19	VD19	MG799240
20	VD20	MG799241
21	VD21	MG799242
22	VD22	MG799243
23	VD23	MG799244
24	VD24	MG799245
25	VD25	MG799246
26	VD26	MG799247
27	VD27	MG799248
28	VD28	MG799249
29	VD29	MG799250
30	VD30	MG799251
31	VD31	MG799252
32	VD32	MG799253
33	VD33	MG799254
34	VD34	MG799255
35	VD35	MG799256
36	VD36	MG799257
37	VD37	MG799258
38	VD38	MG799259
39	VD39	MG799260
40	VD40	MG799261
41	VD41	MG799262
42	VD42	MG799263
43	VD43	MG799264
44	VD44	MG799265
45	VD45	MG799266
46	VD46	MG799267
47	VD47	MG799268
48	VD48	MG799269
49	VD49	MG799270
50	VD50	MG799271
51	VD51	MG799272
52	VD52	MG799273
53	VD53	MG799274
54	VD54	MG799275
55	VD55	MG799276
56	VD56	MG799277
57	VD57	MG799278
58	VD58	MG799279
59	VD59	MG799280
60	VD60	MG799281
61	VD61	MG799282
62	VD62	MG799283
63	VD63	MG799284
64	VD64	MG799285
65	VD65	MG799286
66	VD66	MG799287
67	VD67	MG799288
68	VD68	MG799289
69	VD69	MG799290
70	VD70	MG799291
71	VD71	MG799292
72	VD72	MG799293
73	VD73	MG799294
74	VD74	MG799295
75	VD75	MG799296
76	VD76	MG799297
77	VD77	MG799298
78	VD78	MG799299
79	VD79	MG799300
80	VD80	MG799301
81	VD81	MG799302
82	VD82	MG799303
83	VD83	MG799304
84	VD84	MG799305
85	VD85	MG799306
86	VD86	MG799307
87	VD87	MG799308
88	VD88	MG799309
89	VD89	MG799310
90	VD90	MG799311
91	VD91	MG799312
92	VD92	MG799313
93	VD93	MG799314
94	VD94	MG799315
95	VD95	MG799316
96	VD96	MG799317
97	VD97	MG799318
98	VD98	MG799319
99	VD99	MG799320
100	VD100	MG799321
101	PQ1	MG793253
102	PQ2	MG793254

No.	Sample	GenBank accession number
103	PQ3	MG793255
104	PQ5	MG793256
105	PQ6	MG793257
106	PQ11	MG793258
107	PQ12	MG793259
108	PQ13	MG793260
109	PQ14	MG793261
110	PQ15	MG793262
111	PQ16	MG793263
112	PQ17	MG793264
113	PQ18	MG793265
114	PQ19	MG793266
115	PQ20	MG793267
116	PQ21	MG793268
117	PQ22	MG793269
118	PQ23	MG793270
119	PQ24	MG793271
120	PQ25	MG793272
121	PQ26	MG793273
122	PQ28	MG793274
123	PQ30	MG793275
124	PQ31	MG793276
125	PQ32	MG793277
126	PQ33	MG793278
127	PQ34	MG793279
128	PQ35	MG793280
129	PQ36	MG793281
130	PQ37	MG793282
131	PQ38	MG793283
132	PQ39	MG793284
133	PQ40	MG793285
134	PQ41	MG793286
135	PQ42	MG793287
136	PQ43	MG793288
137	PQ44	MG793289
138	PQ45	MG793290
139	PQ46	MG793291
140	PQ47	MG793292
141	PQ48	MG793293
142	PQ49	MG793294
143	PQ50	MG793295
144	PQ51	MG793296
145	PQ52	MG793297
146	PQ53	MG793298
147	PQ54	MG793299
148	PQ55	MG793300
149	PQ56	MG793301
150	PQ57	MG793302
151	PQ58	MG793303
152	PQ59	MG793304
153	PQ60	MG793305
154	PQ61	MG793306
155	PQ62	MG793307
156	PQ63	MG793308
157	PQ64	MG793309
158	PQ65	MG793310
159	PQ66	MG793311
160	PQ67	MG793312
161	PQ68	MG793313
162	PQ69	MG793314
163	PQ70	MG793315
164	PQ71	MG793316
165	PQ72	MG793317
166	PQ73	MG793318
167	PQ74	MG793319
168	PQ75	MG793320
169	PQ76	MG793321
170	PQ77	MG793322
171	PQ78	MG793323
172	PQ79	MG793324
173	PQ80	MG793325
174	PQ81	MG793326
175	PQ82	MG793327
176	PQ83	MG793328
177	PQ84	MG793329
178	PQ85	MG793330
179	PQ86	MG793331
180	PQ87	MG793332
181	PQ88	MG793333
182	PQ89	MG793334
183	PQ90	MG793335
184	PQ91	MG793336
185	PQ92	MG793337
186	PQ93	MG793338
187	PQ94	MG793339
188	PQ95	MG793340
189	PQ96	MG793341
190	PQ97	MG793342
191	PQ98	MG793343
192	PQ99	MG793344
193	PQ100	MG793345
194	PQ101	MG793346
195	PQ102	MG793347
196	PQ103	MG793348
197	PQ104	MG793349
198	PQ105	MG793350
199	PQ106	MG793351
200	PQ107	MG793352

No.	Sample	GenBank accession number
201	VDBH1	OQ241527
202	VDBH2	OQ241528
203	VDBH3	OQ241529
204	VDBH4	OQ241530
205	VDBH5	OQ241531
206	VDBH6	OQ241532
207	VDBH7	OQ241533
208	VDBH8	OQ241534
209	VDBH9	OQ241535
210	VDBH10	OQ241536
211	VDBH11	OQ241537
212	VDBH12	OQ241538
213	VDBH13	OQ241539
214	VDBH14	OQ241540
215	VDBH15	OQ241541
216	VDST1	OQ241542
217	VDST2	OQ241543
218	VDST3	OQ241544
219	VDST4	OQ241545
220	VDST5	OQ241546
221	VDST6	OQ241547
222	VDST7	OQ241548
223	VDST8	OQ241549
224	VDST9	OQ241550
225	VDST10	OQ241551
226	VDST11	OQ241552
227	VDST12	OQ241553
228	VDST13	OQ241554
229	VDST14	OQ241555
230	VDST15	OQ241556
231	VDST16	OQ241557
232	VDST17	OQ241558
233	VDST18	OQ241559
234	VDST19	OQ241560

No.	Sample	GenBank accession number
235	VDST20	OQ241561
236	VDST21	OQ241562
237	VDRG1	OQ241563
238	VDRG2	OQ241564
239	VDRG3	OQ241565
240	VDRG4	OQ241566
241	VDRG5	OQ241567
242	VDRG6	OQ241568
243	VDRG7	OQ241569
244	VDRG8	OQ241570
245	VDRG9	OQ241571
246	VDRG10	OQ241572
247	VDRG11	OQ241573
248	VDRG12	OQ241574
249	VDRG13	OQ241575
250	VDRG14	OQ241576
251	VDRG15	OQ241577
252	VDRG16	OQ241578
253	VDRG17	OQ241579
254	VDRG18	OQ241580
255	VDRG19	OQ241581
256	VDRG20	OQ241582
257	VDRG21	OQ241583
258	VDRG22	OQ241584
259	VDRG23	OQ241585
260	VDRG24	OQ241586
261	VDRG25	OQ241587
262	VDRG26	OQ241588
263	VDRG27	OQ241589
264	VDRG28	OQ241590
265	VDRG29	OQ241591
266	VDRG30	OQ241592
267	VDRG31	OQ241593
268	VDRG32	OQ241594

No.	Sample	GenBank accession number
269	VDRG33	OQ241595
270	VDRG34	OQ241596
271	VDRG35	OQ241597
272	VDRG36	OQ241598
273	VDRG37	OQ241599
274	VDRG38	OQ241600
275	VDRG39	OQ241601
276	VDRG40	OQ241602
277	VDRG41	OQ241603
278	VDRG42	OQ241604
279	VDRG43	OQ241605
280	VDRG44	OQ241606
281	VDRG45	OQ241607
282	VDRG46	OQ241608
283	VDRG47	OQ241609
284	VDRG48	OQ241610
285	VDRG49	OQ241611
286	VDRG50	OQ241612
287	VDHT1	OQ241613
288	VDHT2	OQ241614
289	VDHT3	OQ241615
290	VDHT4	OQ241616
291	VDHT5	OQ241617
292	VDHT6	OQ241618
293	VDHT7	OQ241619
294	VDHT8	OQ241620
295	VDHT9	OQ241621
296	VDHT10	OQ241622
297	VDHT11	OQ241623
298	VDHT12	OQ241624
299	VDHT13	OQ241625
300	VDHT14	OQ241626
301	VDHT15	OQ241627
302	VDHM1	OQ241628

No.	Sample	GenBank accession number
303	VDHM2	OQ241629
304	VDHM3	OQ241630
305	VDHM4	OQ241631
306	VDHM5	OQ241632
307	VDHM6	OQ241633
308	VDHM7	OQ241634
309	VDHM8	OQ241635
310	VDHM9	OQ241636
311	VDHM10	OQ241637
312	VDHM11	OQ241638
313	VDHM12	OQ241639
314	VDHM13	OQ241640
315	VDHM14	OQ241641
316	VDHM15	OQ241642
317	VDHM16	OQ241643
318	VDHM17	OQ241644
319	VDHM18	OQ241645
320	VDHM19	OQ241646
321	VDHM20	OQ241647
322	VDHM21	OQ241648
323	VDHM22	OQ241649
324	VDHM23	OQ241650
325	VDHM24	OQ241651
326	VDHM25	OQ241652
327	VDHM26	OQ241653
328	VDHM27	OQ241654
329	VDHM28	OQ241655
330	VDHM29	OQ241656
331	VDHM30	OQ241657
332	VDHM31	OQ241658
333	VDPQ1	OQ241659
334	VDPQ2	OQ241660
335	VDPQ3	OQ241661
336	VDPQ4	OQ241662

No.	Sample	GenBank accession number
337	VDPQ5	OQ241663
338	VDPQ6	OQ241664
339	VDPQ7	OQ241665
340	VDPQ8	OQ241666
341	VDPQ9	OQ241667
342	VDPQ10	OQ241668
343	VDPQ11	OQ241669
344	VDPQ12	OQ241670
345	VDPQ13	OQ241671
346	VDPQ14	OQ241672
347	VDPQ15	OQ241673
348	VDPQ16	OQ241674
349	VDPQ17	OQ241675
350	VDPQ18	OQ241676
351	VDPQ19	OQ241677
352	VDPQ20	OQ241678
353	VDPQ21	OQ241679
354	VDPQ22	OQ241680
355	VDPQ23	OQ241681
356	VDPQ24	OQ241682
357	VDPQ25	OQ241683
358	VDPQ26	OQ241684
359	VDPQ27	OQ241685
360	VDPQ28	OQ241686
361	VDPQ29	OQ241687
362	VDPQ30	OQ241688
363	VDPQ31	OQ241689
364	VDPQ32	OQ241690
365	VDPQ33	OQ241691
366	VDPQ34	OQ241692
367	VDPQ35	OQ241693
368	VDPQ36	OQ241694
369	VDPQ37	OQ241695
370	VDPQ38	OQ241696

No.	Sample	GenBank accession number
371	VDPQ39	OQ241697
372	VDPQ40	OQ241698
373	VDPQ41	OQ241699
374	VDPQ42	OQ241700
375	VDPQ43	OQ241701
376	VDPQ44	OQ241702
377	VDPQ45	OQ241703
378	VDPQ46	OQ241704
379	VDPQ47	OQ241705
380	VDPQ48	OQ241706
381	VDPQ49	OQ241707
382	VDPQ50	OQ241708
383	VDHU1	OQ241709
384	VDHU2	OQ241710
385	VDHU3	OQ241711
386	VDHU4	OQ241712
387	VDHU5	OQ241713
388	VDHU6	OQ241714
389	VDHU7	OQ241715
390	VDHU8	OQ241716
391	VDHU9	OQ241717
392	VDHU10	OQ241718
393	VDHU11	OQ241719
394	VDHU12	OQ241720
395	VDHU13	OQ241721
396	VDHU14	OQ241722
397	VDHU15	OQ241723
